# Editorial: Midlife brain health: understanding brain aging in middle-age and effects of interventions to prevent neurodegeneration in late life

**DOI:** 10.3389/fnagi.2025.1568500

**Published:** 2025-02-19

**Authors:** Junyeon Won, Marissa A. Gogniat, Takuya Kurazumi, Kristy A. Nielson

**Affiliations:** ^1^Department of Neurology, University of Texas Southwestern Medical Center, Dallas, TX, United States; ^2^Institute for Exercise and Environmental Medicine, Texas Health Presbyterian Hospital, Dallas, TX, United States; ^3^Department of Neurology, University of Pittsburgh School of Medicine, Pittsburgh, PA, United States; ^4^Department of Social Medicine, Division of Hygiene, Nihon University School of Medicine, Tokyo, Japan; ^5^Department of Psychology, Marquette University, Milwaukee, WI, United States; ^6^Department of Neurology, Medical College of Wisconsin, Milwaukee, WI, United States

**Keywords:** aging, middle-age, neurodegeneration, Alzheimer's disease, exercise, memory

## 1 Introduction

With the rapid increase in the aging population, the prevalence of age-related neurodegenerative diseases such as Alzheimer's disease (AD) has risen significantly, affecting over 55 million people worldwide in 2023, with projections suggesting this number will exceed 78 million by 2030 (Better, 2023). While much research has been focused on understanding and treating AD in older adults, there is growing emphasis on early interventions to prevent its onset (Crous-Bou et al., 2017; Dohm-Hansen et al., 2024). In this regard, middle-age has gained recognition as a critical period for the development and prevention of AD (Ritchie et al., 2017). For example, midlife vascular risk factors including diabetes, obesity, and hypertension increased the risk of developing AD in late life (Livingston et al., 2020). This Research Topic includes eight human and rodent studies, including three review papers, exploring strategies to reduce the risk of age-related cognitive decline and mechanisms of age-related memory decline in middle-aged individuals, older adults with normal cognition, those with mild cognitive impairment (MCI), and those with AD.

## 2 Exercise intervention

### 2.1 Normal cognition

In their review, Eubank et al. highlighted that lifestyle factors and interventions, including physical activity, dietary intake, sleep, and stress management, may help prevent or delay age-related cognitive decline. Among these, physical activity and dietary intake showed the strongest associations with cognitive health. However, the study also identified a significant gap in the current literature regarding underserved populations, emphasizing the need for more targeted research and interventions in these groups (Eubank et al.).

Gujral et al. examined the effects of exercise training on cognitive function in cognitively normal middle-aged and older adults (total *n* = 25; aerobic exercise *n* = 15; low intensity movement training *n* = 10). They found that exercise intensity influenced specific cognitive domains. Low-intensity movement exercise enhanced learning and memory, while moderate-intensity aerobic exercise improved executive functioning. Both exercise interventions led to an increase in the surface area of the right dorsolateral prefrontal cortex, which correlated with improvements in executive function (Gujral et al.). However, because this study employed a relatively small number of participants who are mostly white and highly-educated, futures studies with larger sample size with diverse sample characteristics are needed to confirm the findings.

### 2.2 Mild-cognitive impairment

Huang et al. reviewed studies on older adults with mild cognitive impairment (MCI), a prodromal stage of AD. Although MCI can be a precursor to development of AD, many individuals with MCI remain cognitively stable and may even show improvements in cognitive performance, making MCI a critical window for implementing non-pharmacological interventions to delay neurological deterioration. The study explored hypothesized molecular mechanisms underlying the protective effects of aerobic exercise against MCI. Aerobic exercise reduces cardiovascular risk factors, such as hypertension and type 2 diabetes mellitus, which significantly elevate the risk of MCI. On a molecular level, aerobic exercise is thought to modulate the activation of microglia and astrocytes, key players in brain inflammation and neurogenesis. Additionally, it may enhance synaptic plasticity and provide neuroprotection through the release of exercise-induced factors like irisin and cathepsin B (Huang et al.).

### 2.3 Alzheimer's disease

In a systematic review, Yi et al. examined the effects of virtual reality (VR) exercise intervention on cognitive function in patients with AD. Their findings indicated that VR interventions improved cognition, memory, executive function, and body balance in these patients. Considering the logistical challenges AD patients face in participating in group exercises, this review highlights the potential of VR therapies as an effective tool for cognitive rehabilitation and enhancing physical function (Yi et al.).

Using an AD mouse model, Shi et al. demonstrated that both chlorogenic acid administration and moderate-intensity aerobic exercise independently improved oxidative stress, neuroinflammation, Amyloid Beta (Aβ) deposition, and cognitive performance. While no significant differences were observed between the effects of each intervention alone, the combination of chlorogenic acid and aerobic exercise produced greater reductions in AD biomarkers, suggesting synergistic benefits (Shi et al.).

## 3 Memory

### 3.1 Spatial memory

Puthusseryppady et al. examined spatial exploration behavior during the normal aging process. A total of 55 healthy young adults (18–28 years) and 87 healthy middle-aged adults (43–61 years) completed a desktop virtual maze task, during which their exploration behaviors were assessed. In the test phase, participants navigated between target objects without feedback, and their wayfinding success rate (percentage of correct trials) was measured. The results revealed that middle-aged adults engaged in less exploration compared to young adults during the exploration phase and demonstrated lower success rates during the test phase. Among middle-aged adults, both the quantity and quality of exploration were linked to wayfinding success. These findings highlight the effects of aging on spatial exploration behavior in middle-aged individuals (Puthusseryppady et al.).

### 3.2 External factors affecting perception of memory and memory network

Paban et al. sought to identify cognitive and psychological contributing factors and characterize cerebral hubs within the brain network in older adults with subjective cognitive decline (SCD). A total of 45 older adults with normal cognition and 50 older adults with SCD completed a series of neuropsychological tests, psychological questionnaires, and electroencephalogram (EEG) recordings. The study found that lower self-esteem and conscientiousness scores were associated with higher levels of SCD. Additionally, network topography in the delta and theta frequency bands differed significantly between the SCD and non-SCD groups. Notably, in individuals with SCD, the inferior temporal gyrus and left orbitofrontal area emerged as new network hubs, while the dorsolateral prefrontal cortex and middle temporal gyrus lost their hub functions. These findings suggest that personality factors may play a critical role in SCD and that SCD can alter the hub roles of specific brain regions (Paban et al.).

### 3.3 Mechanisms at the synaptic level

Zheng et al. explored the role of activin A in synaptic plasticity during normal aging using mouse models. They observed that hippocampi from older mice exhibited higher levels of both activin A and its receptor compared to those from young mice. Notably, in middle-aged hippocampi, there was a significant increase in activin A and endogenous activin receptor IB levels. Furthermore, administering recombinant activin A successfully restored full long-term potentiation (LTP) in slices from young dominant-negative mutant activin receptor IB (dnActRIB) mice. These findings suggest that endogenous activin receptor signaling strengthens beginning in midlife, potentially mitigating age-related cognitive decline (Zheng et al.).

## 4 Conclusion

The studies included in this Research Topic highlight the effects of exercise training on cognitive and brain health across various stages of cognitive function—from middle-aged individuals to older adults with MCI and AD. Additionally, other research demonstrates that aging impacts memory through changes in spatial exploration, brain network dynamics, and synaptic mechanisms, with factors such as personality traits and molecular signaling as well as vascular health playing critical roles in cognitive decline and adaptation (Gogniat et al., 2020; Won et al., 2025a,b). Moving forward, future research should focus on elucidating the underlying mechanisms that link midlife risk factors to late-life cognitive outcomes.
